# Bis(μ-4,4′′-difluoro-1,1′:3′,1′′-terphenyl-2′-carboxylato-κ^2^ *O*:*O*′)bis[aqua(4,4′′-di­fluoro-1,1′:3′,1′′-terphenyl-2′-carboxyl­ato-κ*O*)(pyridine-κ*N*)cobalt(II)] diethyl ether disolvate

**DOI:** 10.1107/S1600536812038391

**Published:** 2012-09-15

**Authors:** Namseok Kim, Yeahsel Yoon, Ha-Jin Lee, Sungho Yoon

**Affiliations:** a Department of Bio & Nano Chemistry, College of Natural Sciences, Kookmin University, 861-1 Jeongneung-dong, Seongbuk-gu, Seoul 136-702, Republic of Korea; b Jeonju Center, Korea Basic Science Institute (KBSI), 664-14 Dukjin dong 1-ga, Dukjin-gu, Jeonju 561-756, Republic of Korea

## Abstract

The structure of the title compound, [Co_2_(C_19_H_11_F_2_O_2_)_4_(C_5_H_5_N)_2_(H_2_O)_2_]·2C_4_H_10_O, comprises two Co^II^ atoms in a distorted square pyramidal coordination environment, straddling a crystallographic inversion center with a Co⋯Co separation of 3.1923 (15) Å. Each Co^2+^ cation is coordinated by three O atoms of three 4,4′′-difluoro-1,1′:3′,1′′-terphenyl-2′-carboxyl­ate ligands, one water O atom and one pyridine N atom, forming a CoO_4_N polyhedron. Strong intra­molecular O—H⋯O hydrogen bonds are observed between terminal metal-bound carboxyl­ate groups and water O atoms.

## Related literature

For background to metal complexes with 4,4′′-difluoro-1,1′:3′,1′′-terphenyl-2′-carboxyl­ate ligands, see: Kannan *et al.* (2011 ▶) and to water-bridged di-cobalt complexes, see: Lee *et al.* (2002 ▶). Bimetal systems, ligated by four carboxyl­ates and two histidines derived from the side chains of amino acids, are often found in metalloenzyme active sites, see: Holm *et al.* (1996 ▶); Lippard & Berg (1994 ▶).
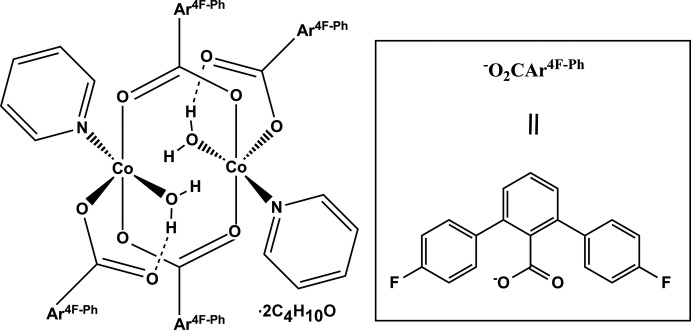


## Experimental

### 

#### Crystal data

[Co_2_(C_19_H_11_F_2_O_2_)_4_(C_5_H_5_N)_2_(H_2_O)_2_]·2C_4_H_10_O
*M* *_r_* = 1697.44
Triclinic, 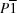

*a* = 12.0347 (16) Å
*b* = 14.0597 (18) Å
*c* = 14.3547 (18) Å
α = 113.199 (3)°
β = 91.182 (3)°
γ = 113.336 (3)°
*V* = 2004.4 (4) Å^3^
*Z* = 1
Mo *K*α radiation
μ = 0.50 mm^−1^
*T* = 200 K
0.24 × 0.16 × 0.10 mm

#### Data collection

Bruker SMART CCD area-detector diffractometer
Absorption correction: multi-scan (*SADABS*; Bruker, 2000 ▶) *T*_min_ = 0.403, *T*_max_ = 1
15130 measured reflections
9861 independent reflections
4923 reflections with *I* > 2σ(*I*)
*R*_int_ = 0.042

#### Refinement

*R*[*F* ^2^ > 2σ(*F* ^2^)] = 0.071
*wR*(*F* ^2^) = 0.239
*S* = 1.06
9861 reflections
539 parameters
H atoms treated by a mixture of independent and constrained refinement
Δρ_max_ = 0.83 e Å^−3^
Δρ_min_ = −1.68 e Å^−3^

### 

Data collection: *SMART* (Bruker, 2000 ▶); cell refinement: *SAINT* (Bruker, 2000 ▶); data reduction: *SAINT*; program(s) used to solve structure: *SHELXS97* (Sheldrick, 2008) ▶; program(s) used to refine structure: *SHELXL97* (Sheldrick, 2008) ▶; molecular graphics: *SHELXTL* (Sheldrick, 2008) ▶; software used to prepare material for publication: *SHELXTL* ▶.

## Supplementary Material

Crystal structure: contains datablock(s) I, global. DOI: 10.1107/S1600536812038391/ru2042sup1.cif

Structure factors: contains datablock(s) I. DOI: 10.1107/S1600536812038391/ru2042Isup2.hkl

Additional supplementary materials:  crystallographic information; 3D view; checkCIF report

## Figures and Tables

**Table 1:** Selected bond lengths (Å)

Co1—O4	2.025 (3)
Co1—O2	2.032 (3)
Co1—O3	2.040 (3)
Co1—N1	2.097 (4)
Co1—O5	2.230 (4)

**Table 2:** Hydrogen-bond geometry (Å, °)

*D*—H⋯*A*	*D*—H	H⋯*A*	*D*⋯*A*	*D*—H⋯*A*
O5—H5⋯O6	0.84	1.87	2.602 (5)	145

**Fig. 1.**
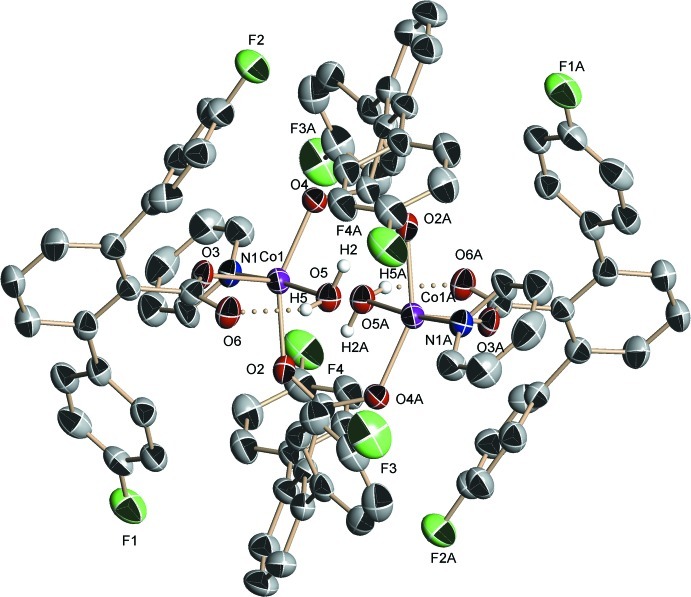
The molecular structure of the title compound, showing the atom-numbering, the hydrogen bonds and with displacement ellipsoids drawn at the 50% probability level.

## supplementary crystallographic information

### Comment

In metalloenzyme active sites, bimetal systems, ligated by four carboxylates and two histidines derived from the side chains of amino acids, are often found (Lippard *et al.* 1994; Holm *et al.* 1996). Here, we report the structure of the water-containing di-nuclear Co(II) complex which crystallizes in the triclinic space group *P*1 with one half molecule in the asymmetric unit. Bond distances to the metal are given in Table 1 with the structure of the molecule shown in Fig 1. and its strong intramolecular O—H···O interactions detailed in Table 2.

### Experimental

The sodium 4,4''-difluoro-1,1':3',1''-terphenyl-2'-carboxylate (0.200 g, 0.602 mmol) was added into cobalt(II) trifluoromethansulfonate (0.529 g, 1.21 mmol) in 10 ml of tetrahydrofuran at room temperature. After stirring for 30 min, triethylamine (0.122 g, 1.21 mmol) and pyridine (0.134 g, 1.69 mmol) were added. Immediately, the color of solution was changed from light violet to dark purple. After 30 min, water (0.0218 g, 1.21 mmol) was further added. The volatile fractions were removed under the reduced pressure. Resulting purple powder was dissolved in dicloromethane and insoluble fractions were filtered off. Purple block-shaped crystals were collected upon vapor diffusion of diethyl ether. Yield = 2.26% (0.0230 g)

### Refinement

H atoms were placed at calculated positions and refined as riding with C–H(aromatic) = 0.95 Å, C–H(CH_3_) = 0.98 Å, and with *U*_iso_(H) = 1.2 *U*_eq_(C) or 1.5 *U*_eq_(C) for methyl groups. The O-bound H atoms of waters were located in a difference Fourier map and refined isotropically.

### Crystal data

**Table:**

[Co_2_(C_19_H_11_F_2_O_2_)_4_(C_5_H_5_N)_2_(H_2_O)_2_]·2C_4_H_10_O	*Z* = 1
*M**_r_* = 1697.44	*F*(000) = 878
Triclinic, *P*1	*D*_x_ = 1.406 Mg m^−^^3^
Hall symbol: -P 1	Mo *K*α radiation, λ = 0.71073 Å
*a* = 12.0347 (16) Å	Cell parameters from 3108 reflections
*b* = 14.0597 (18) Å	θ = 2.2–24.3°
*c* = 14.3547 (18) Å	µ = 0.50 mm^−^^1^
α = 113.199 (3)°	*T* = 200 K
β = 91.182 (3)°	Block, pink
γ = 113.336 (3)°	0.24 × 0.16 × 0.10 mm
*V* = 2004.4 (4) Å^3^	

### Data collection

**Table:**

Bruker SMART CCD area-detector diffractometer	9861 independent reflections
Radiation source: sealed tube	4923 reflections with *I* > 2σ(*I*)
Graphite monochromator	*R*_int_ = 0.042
phi and ω scans	θ_max_ = 28.4°, θ_min_ = 1.6°
Absorption correction: multi-scan (*SADABS*; Bruker, 2000)	*h* = −16→15
*T*_min_ = 0.403, *T*_max_ = 1	*k* = −18→17
15130 measured reflections	*l* = −14→19

### Refinement

**Table:**

Refinement on *F*^2^	Primary atom site location: structure-invariant direct methods
Least-squares matrix: full	Secondary atom site location: difference Fourier map
*R*[*F*^2^ > 2σ(*F*^2^)] = 0.071	Hydrogen site location: inferred from neighbouring sites
*wR*(*F*^2^) = 0.239	H atoms treated by a mixture of independent and constrained refinement
*S* = 1.06	*w* = 1/[σ^2^(*F*_o_^2^) + (0.0806*P*)^2^ + 3.4487*P*] where *P* = (*F*_o_^2^ + 2*F*_c_^2^)/3
9861 reflections	(Δ/σ)_max_ < 0.001
539 parameters	Δρ_max_ = 0.83 e Å^−^^3^
0 restraints	Δρ_min_ = −1.68 e Å^−^^3^

### Special details

**Table:**

Geometry. All e.s.d.'s (except the e.s.d. in the dihedral angle between two l.s. planes) are estimated using the full covariance matrix. The cell e.s.d.'s are taken into account individually in the estimation of e.s.d.'s in distances, angles and torsion angles; correlations between e.s.d.'s in cell parameters are only used when they are defined by crystal symmetry. An approximate (isotropic) treatment of cell e.s.d.'s is used for estimating e.s.d.'s involving l.s. planes.
Refinement. Refinement of *F*^2^ against ALL reflections. The weighted *R*-factor *wR* and goodness of fit *S* are based on *F*^2^, conventional *R*-factors *R* are based on *F*, with *F* set to zero for negative *F*^2^. The threshold expression of *F*^2^ > σ(*F*^2^) is used only for calculating *R*-factors(gt) *etc*. and is not relevant to the choice of reflections for refinement. *R*-factors based on *F*^2^ are statistically about twice as large as those based on *F*, and *R*- factors based on ALL data will be even larger.

### Fractional atomic coordinates and isotropic or equivalent isotropic displacement parameters (Å^2^)

**Table:**

	*x*	*y*	*z*	*U*_iso_*/*U*_eq_	
Co1	0.60580 (6)	0.59297 (6)	0.60114 (5)	0.0361 (2)	
O5	0.4457 (3)	0.4290 (3)	0.5772 (3)	0.0395 (8)	
H5	0.4256	0.4347	0.6342	0.059*	
O2	0.4692 (3)	0.6439 (3)	0.6041 (2)	0.0379 (8)	
N1	0.7461 (4)	0.7539 (3)	0.6268 (3)	0.0371 (9)	
O3	0.6498 (3)	0.6358 (3)	0.7548 (2)	0.0387 (8)	
O4	0.6902 (3)	0.4936 (3)	0.5279 (2)	0.0381 (8)	
C1	0.3618 (5)	0.6013 (4)	0.5535 (4)	0.0354 (11)	
C29	0.8383 (5)	0.7648 (5)	0.5777 (4)	0.0516 (14)	
H29	0.8413	0.6972	0.5283	0.062*	
O6	0.4785 (3)	0.5124 (3)	0.7781 (3)	0.0457 (9)	
C25	0.2179 (5)	0.4920 (5)	0.6837 (4)	0.0466 (13)	
H25	0.3031	0.5443	0.7098	0.056*	
C22	0.2740 (6)	0.8489 (5)	0.6312 (4)	0.0545 (15)	
H22	0.3024	0.9217	0.6300	0.065*	
C24	0.1389 (5)	0.5206 (5)	0.6390 (4)	0.0422 (12)	
C19	0.1880 (5)	0.6339 (5)	0.6338 (4)	0.0462 (13)	
C3	0.5839 (5)	0.5962 (4)	0.8106 (4)	0.0369 (11)	
C10	0.6781 (5)	0.4879 (5)	0.9112 (4)	0.0410 (12)	
C26	0.4369 (5)	0.8181 (5)	0.5376 (4)	0.0452 (13)	
C21	0.3327 (5)	0.7785 (5)	0.5877 (4)	0.0440 (13)	
C4	0.6390 (4)	0.6573 (4)	0.9245 (4)	0.0365 (11)	
C8	0.7213 (5)	0.6549 (5)	1.0786 (4)	0.0528 (15)	
H8	0.7450	0.6165	1.1109	0.063*	
C13	0.7536 (5)	0.4762 (5)	0.8393 (4)	0.0504 (14)	
H13	0.7966	0.5380	0.8220	0.061*	
C7	0.7322 (6)	0.7639 (5)	1.1381 (4)	0.0543 (15)	
H7	0.7640	0.8000	1.2107	0.065*	
C14	0.6089 (5)	0.8327 (4)	0.9435 (4)	0.0421 (12)	
C18	0.4481 (6)	0.8445 (5)	0.8503 (4)	0.0503 (14)	
H18	0.3672	0.8101	0.8097	0.060*	
C17	0.6448 (6)	1.0092 (5)	0.9324 (5)	0.0564 (15)	
H17	0.6983	1.0869	0.9478	0.068*	
C5	0.6501 (5)	0.7689 (5)	0.9859 (4)	0.0413 (12)	
C20	0.2899 (5)	0.6711 (4)	0.5899 (4)	0.0387 (12)	
C9	0.6757 (5)	0.6000 (4)	0.9712 (4)	0.0402 (12)	
C16	0.6841 (5)	0.9464 (5)	0.9671 (4)	0.0504 (14)	
H16	0.7650	0.9826	1.0083	0.060*	
C15	0.4889 (5)	0.7821 (5)	0.8853 (4)	0.0455 (13)	
H15	0.4347	0.7045	0.8696	0.055*	
C12	0.6227 (6)	0.2921 (5)	0.8832 (5)	0.0562 (15)	
H12	0.5763	0.2278	0.8964	0.067*	
C23	0.1297 (6)	0.7061 (6)	0.6760 (4)	0.0572 (16)	
H23	0.0593	0.6815	0.7047	0.069*	
C11	0.6128 (5)	0.3943 (5)	0.9315 (5)	0.0519 (14)	
H11	0.5602	0.4005	0.9797	0.062*	
C6	0.6975 (5)	0.8205 (5)	1.0934 (4)	0.0489 (14)	
H6	0.7054	0.8956	1.1354	0.059*	
C28	0.7429 (5)	0.8496 (4)	0.6971 (4)	0.0505 (14)	
H28	0.6764	0.8423	0.7325	0.061*	
F1	0.4869 (4)	1.0186 (3)	0.8430 (3)	0.0703 (10)	
F3	0.0045 (4)	0.2143 (3)	0.6617 (3)	0.0938 (14)	
F2	0.7149 (4)	0.1870 (3)	0.7706 (3)	0.0806 (12)	
C45	0.1733 (6)	0.3890 (5)	0.6903 (4)	0.0535 (15)	
H45	0.2273	0.3682	0.7185	0.064*	
C39	0.0140 (5)	0.4445 (6)	0.6039 (4)	0.0536 (15)	
H39	−0.0409	0.4638	0.5750	0.064*	
C34	0.4377 (6)	0.7469 (5)	0.4357 (4)	0.0508 (14)	
H34	0.3704	0.6728	0.3985	0.061*	
C44	0.5280 (6)	0.9570 (5)	0.8759 (4)	0.0499 (14)	
C41	0.0492 (7)	0.3168 (5)	0.6554 (5)	0.0603 (17)	
C38	0.5357 (6)	0.7841 (5)	0.3893 (4)	0.0565 (16)	
H38	0.5355	0.7364	0.3204	0.068*	
C42	0.7665 (6)	0.3747 (5)	0.7925 (4)	0.0581 (16)	
H42	0.8199	0.3672	0.7451	0.070*	
C40	−0.0312 (6)	0.3408 (6)	0.6105 (5)	0.0632 (17)	
H40	−0.1162	0.2875	0.5846	0.076*	
C36	0.6355 (6)	0.9644 (5)	0.5419 (5)	0.0586 (16)	
H36	0.7029	1.0389	0.5771	0.070*	
C37	0.6318 (7)	0.8897 (5)	0.4439 (5)	0.0579 (16)	
C43	0.7007 (6)	0.2865 (5)	0.8166 (5)	0.0547 (15)	
C33	0.1753 (6)	0.8128 (6)	0.6754 (5)	0.0643 (18)	
H33	0.1374	0.8622	0.7064	0.077*	
C35	0.5366 (6)	0.9270 (5)	0.5876 (5)	0.0577 (16)	
H35	0.5370	0.9775	0.6553	0.069*	
C32	0.9283 (6)	0.9697 (6)	0.6685 (6)	0.0684 (19)	
H32	0.9912	1.0440	0.6831	0.082*	
C31	0.8321 (6)	0.9566 (5)	0.7190 (5)	0.0620 (17)	
H31	0.8275	1.0226	0.7696	0.074*	
C30	0.9310 (6)	0.8715 (6)	0.5956 (5)	0.0635 (17)	
H30	0.9957	0.8769	0.5582	0.076*	
F4	0.7304 (4)	0.9255 (3)	0.3996 (3)	0.0778 (11)	
O7	0.0379 (4)	0.7330 (4)	0.9186 (3)	0.0700 (12)	
C47	−0.0130 (6)	0.8100 (6)	0.9638 (5)	0.0666 (18)	
H47A	0.0462	0.8774	1.0263	0.080*	
H47B	−0.0898	0.7718	0.9848	0.080*	
C46	−0.0415 (7)	0.8488 (6)	0.8852 (5)	0.078 (2)	
H46A	0.0350	0.8869	0.8650	0.117*	
H46B	−0.0772	0.9029	0.9159	0.117*	
H46C	−0.1006	0.7817	0.8238	0.117*	
C48	0.0575 (7)	0.6834 (6)	0.9825 (5)	0.075 (2)	
H48A	−0.0216	0.6417	0.9989	0.090*	
H48B	0.1163	0.7441	1.0483	0.090*	
C49	0.1097 (7)	0.6012 (7)	0.9234 (6)	0.083 (2)	
H49A	0.0526	0.5437	0.8570	0.125*	
H49B	0.1204	0.5622	0.9641	0.125*	
H49C	0.1899	0.6441	0.9109	0.125*	
H2	0.461 (8)	0.368 (8)	0.558 (7)	0.13 (3)*	

### Atomic displacement parameters (Å^2^)

**Table:**

	*U*^11^	*U*^22^	*U*^33^	*U*^12^	*U*^13^	*U*^23^
Co1	0.0396 (4)	0.0341 (4)	0.0313 (3)	0.0161 (3)	0.0051 (3)	0.0114 (3)
O5	0.037 (2)	0.036 (2)	0.0378 (19)	0.0103 (17)	0.0062 (16)	0.0154 (16)
O2	0.041 (2)	0.0329 (18)	0.0312 (17)	0.0115 (16)	−0.0016 (15)	0.0108 (15)
N1	0.038 (2)	0.038 (2)	0.032 (2)	0.013 (2)	0.0040 (18)	0.0151 (19)
O3	0.041 (2)	0.047 (2)	0.0231 (16)	0.0160 (17)	0.0015 (14)	0.0142 (15)
O4	0.042 (2)	0.039 (2)	0.0347 (18)	0.0213 (17)	0.0067 (15)	0.0138 (16)
C1	0.049 (3)	0.030 (3)	0.029 (2)	0.018 (2)	0.008 (2)	0.015 (2)
C29	0.051 (3)	0.048 (3)	0.050 (3)	0.020 (3)	0.014 (3)	0.018 (3)
O6	0.037 (2)	0.042 (2)	0.042 (2)	0.0055 (17)	0.0018 (16)	0.0152 (17)
C25	0.046 (3)	0.052 (3)	0.036 (3)	0.019 (3)	0.005 (2)	0.016 (3)
C22	0.063 (4)	0.053 (4)	0.055 (3)	0.033 (3)	0.012 (3)	0.024 (3)
C24	0.047 (3)	0.047 (3)	0.027 (2)	0.020 (3)	0.014 (2)	0.011 (2)
C19	0.046 (3)	0.049 (3)	0.036 (3)	0.024 (3)	0.002 (2)	0.010 (2)
C3	0.043 (3)	0.042 (3)	0.031 (2)	0.020 (3)	0.007 (2)	0.020 (2)
C10	0.036 (3)	0.044 (3)	0.039 (3)	0.016 (2)	0.004 (2)	0.015 (2)
C26	0.065 (4)	0.037 (3)	0.036 (3)	0.030 (3)	0.005 (3)	0.011 (2)
C21	0.059 (3)	0.045 (3)	0.037 (3)	0.033 (3)	0.011 (3)	0.016 (2)
C4	0.035 (3)	0.036 (3)	0.034 (2)	0.015 (2)	0.009 (2)	0.011 (2)
C8	0.061 (4)	0.066 (4)	0.033 (3)	0.030 (3)	0.008 (3)	0.022 (3)
C13	0.050 (3)	0.054 (4)	0.042 (3)	0.020 (3)	0.011 (3)	0.018 (3)
C7	0.062 (4)	0.058 (4)	0.031 (3)	0.028 (3)	0.006 (3)	0.007 (3)
C14	0.047 (3)	0.031 (3)	0.039 (3)	0.014 (2)	0.009 (2)	0.010 (2)
C18	0.064 (4)	0.049 (3)	0.040 (3)	0.034 (3)	0.007 (3)	0.014 (3)
C17	0.064 (4)	0.037 (3)	0.068 (4)	0.022 (3)	0.028 (3)	0.023 (3)
C5	0.040 (3)	0.045 (3)	0.032 (3)	0.013 (2)	0.011 (2)	0.016 (2)
C20	0.045 (3)	0.047 (3)	0.030 (2)	0.027 (3)	0.004 (2)	0.016 (2)
C9	0.040 (3)	0.044 (3)	0.037 (3)	0.019 (2)	0.012 (2)	0.017 (2)
C16	0.048 (3)	0.039 (3)	0.050 (3)	0.014 (3)	0.015 (3)	0.011 (3)
C15	0.050 (3)	0.037 (3)	0.043 (3)	0.017 (3)	0.005 (3)	0.013 (2)
C12	0.056 (4)	0.048 (4)	0.067 (4)	0.026 (3)	0.013 (3)	0.025 (3)
C23	0.054 (4)	0.066 (4)	0.054 (3)	0.037 (3)	0.010 (3)	0.018 (3)
C11	0.049 (3)	0.056 (4)	0.062 (4)	0.025 (3)	0.019 (3)	0.034 (3)
C6	0.055 (3)	0.049 (3)	0.035 (3)	0.022 (3)	0.007 (3)	0.013 (3)
C28	0.056 (4)	0.029 (3)	0.047 (3)	0.016 (3)	0.008 (3)	0.002 (2)
F1	0.111 (3)	0.061 (2)	0.065 (2)	0.055 (2)	0.026 (2)	0.0334 (19)
F3	0.112 (4)	0.066 (3)	0.101 (3)	0.022 (3)	0.042 (3)	0.051 (3)
F2	0.091 (3)	0.055 (2)	0.084 (3)	0.044 (2)	0.009 (2)	0.008 (2)
C45	0.060 (4)	0.057 (4)	0.045 (3)	0.023 (3)	0.018 (3)	0.027 (3)
C39	0.042 (3)	0.074 (4)	0.040 (3)	0.024 (3)	0.015 (3)	0.020 (3)
C34	0.075 (4)	0.045 (3)	0.038 (3)	0.032 (3)	0.005 (3)	0.018 (3)
C44	0.076 (4)	0.047 (3)	0.041 (3)	0.040 (3)	0.024 (3)	0.020 (3)
C41	0.073 (5)	0.049 (4)	0.057 (4)	0.018 (3)	0.029 (3)	0.029 (3)
C38	0.087 (5)	0.049 (4)	0.043 (3)	0.039 (4)	0.016 (3)	0.020 (3)
C42	0.060 (4)	0.058 (4)	0.045 (3)	0.027 (3)	0.015 (3)	0.010 (3)
C40	0.057 (4)	0.062 (4)	0.052 (4)	0.011 (3)	0.021 (3)	0.022 (3)
C36	0.071 (4)	0.043 (3)	0.056 (4)	0.021 (3)	0.015 (3)	0.021 (3)
C37	0.082 (5)	0.053 (4)	0.051 (3)	0.035 (4)	0.024 (3)	0.029 (3)
C43	0.054 (4)	0.044 (3)	0.056 (4)	0.025 (3)	0.003 (3)	0.009 (3)
C33	0.079 (5)	0.068 (4)	0.065 (4)	0.055 (4)	0.017 (4)	0.024 (4)
C35	0.081 (5)	0.047 (4)	0.043 (3)	0.031 (3)	0.009 (3)	0.014 (3)
C32	0.053 (4)	0.047 (4)	0.086 (5)	0.000 (3)	−0.007 (4)	0.034 (4)
C31	0.067 (4)	0.036 (3)	0.065 (4)	0.016 (3)	0.003 (3)	0.013 (3)
C30	0.055 (4)	0.057 (4)	0.074 (4)	0.014 (3)	0.017 (3)	0.035 (4)
F4	0.101 (3)	0.070 (3)	0.078 (3)	0.041 (2)	0.045 (2)	0.043 (2)
O7	0.074 (3)	0.083 (3)	0.062 (3)	0.043 (3)	0.019 (2)	0.033 (3)
C47	0.050 (4)	0.059 (4)	0.067 (4)	0.014 (3)	0.014 (3)	0.015 (3)
C46	0.070 (5)	0.083 (5)	0.071 (5)	0.042 (4)	−0.003 (4)	0.018 (4)
C48	0.061 (4)	0.081 (5)	0.068 (4)	0.020 (4)	0.001 (4)	0.031 (4)
C49	0.077 (5)	0.100 (6)	0.072 (5)	0.047 (5)	0.008 (4)	0.029 (4)

### Geometric parameters (Å, º)

**Table:**

Co1—O4	2.025 (3)	C17—H17	0.9500
Co1—O2	2.032 (3)	C5—C6	1.409 (7)
Co1—O3	2.040 (3)	C16—H16	0.9500
Co1—N1	2.097 (4)	C15—H15	0.9500
Co1—O5	2.230 (4)	C12—C43	1.356 (8)
O5—H5	0.8400	C12—C11	1.383 (8)
O5—H2	0.89 (9)	C12—H12	0.9500
O2—C1	1.250 (6)	C23—C33	1.381 (9)
N1—C29	1.317 (7)	C23—H23	0.9500
N1—C28	1.342 (6)	C11—H11	0.9500
O3—C3	1.267 (6)	C6—H6	0.9500
O4—C1^i^	1.273 (5)	C28—C31	1.360 (8)
C1—O4^i^	1.273 (5)	C28—H28	0.9500
C1—C20	1.507 (7)	F1—C44	1.367 (6)
C29—C30	1.385 (8)	F3—C41	1.366 (7)
C29—H29	0.9500	F2—C43	1.374 (6)
O6—O6	0.000 (8)	C45—C41	1.372 (8)
O6—C3	1.255 (6)	C45—H45	0.9500
C25—C45	1.375 (8)	C39—C40	1.384 (9)
C25—C24	1.400 (7)	C39—H39	0.9500
C25—H25	0.9500	C34—C38	1.387 (8)
C22—C33	1.371 (9)	C34—H34	0.9500
C22—C21	1.398 (7)	C41—C40	1.366 (9)
C22—H22	0.9500	C38—C37	1.357 (9)
C24—C39	1.388 (8)	C38—H38	0.9500
C24—C19	1.498 (8)	C42—C43	1.363 (9)
C19—C20	1.399 (7)	C42—H42	0.9500
C19—C23	1.409 (7)	C40—H40	0.9500
C3—O6	1.255 (6)	C36—C37	1.372 (8)
C3—C4	1.499 (7)	C36—C35	1.389 (9)
C10—C11	1.389 (7)	C36—H36	0.9500
C10—C13	1.394 (7)	C37—F4	1.369 (7)
C10—C9	1.481 (7)	C33—H33	0.9500
C26—C35	1.394 (8)	C35—H35	0.9500
C26—C34	1.413 (7)	C32—C31	1.369 (9)
C26—C21	1.483 (8)	C32—C30	1.379 (9)
C21—C20	1.403 (7)	C32—H32	0.9500
C4—C9	1.411 (7)	C31—H31	0.9500
C4—C5	1.413 (7)	C30—H30	0.9500
C8—C7	1.378 (8)	O7—C47	1.398 (7)
C8—C9	1.401 (7)	O7—C48	1.418 (8)
C8—H8	0.9500	C47—C46	1.519 (9)
C13—C42	1.392 (8)	C47—H47A	0.9900
C13—H13	0.9500	C47—H47B	0.9900
C7—C6	1.370 (8)	C46—H46A	0.9800
C7—H7	0.9500	C46—H46B	0.9800
C14—C16	1.380 (7)	C46—H46C	0.9800
C14—C15	1.399 (7)	C48—C49	1.508 (9)
C14—C5	1.480 (7)	C48—H48A	0.9900
C18—C44	1.373 (8)	C48—H48B	0.9900
C18—C15	1.393 (7)	C49—H49A	0.9800
C18—H18	0.9500	C49—H49B	0.9800
C17—C44	1.356 (8)	C49—H49C	0.9800
C17—C16	1.385 (8)		
			
O4—Co1—O2	151.94 (13)	C43—C12—C11	117.9 (6)
O4—Co1—O3	106.05 (13)	C43—C12—H12	121.0
O2—Co1—O3	99.17 (13)	C11—C12—H12	121.0
O4—Co1—N1	98.68 (15)	C33—C23—C19	119.8 (6)
O2—Co1—N1	93.70 (15)	C33—C23—H23	120.1
O3—Co1—N1	89.24 (14)	C19—C23—H23	120.1
O4—Co1—O5	86.10 (14)	C12—C11—C10	121.6 (6)
O2—Co1—O5	81.46 (14)	C12—C11—H11	119.2
O3—Co1—O5	90.60 (13)	C10—C11—H11	119.2
N1—Co1—O5	175.07 (16)	C7—C6—C5	120.9 (5)
Co1—O5—H5	109.5	C7—C6—H6	119.6
Co1—O5—H2	116 (6)	C5—C6—H6	119.6
H5—O5—H2	101.1	N1—C28—C31	121.9 (6)
C1—O2—Co1	136.2 (3)	N1—C28—H28	119.1
C29—N1—C28	118.7 (5)	C31—C28—H28	119.1
C29—N1—Co1	122.5 (4)	C41—C45—C25	118.4 (6)
C28—N1—Co1	118.8 (4)	C41—C45—H45	120.8
C3—O3—Co1	129.3 (3)	C25—C45—H45	120.8
C1^i^—O4—Co1	124.6 (3)	C40—C39—C24	120.7 (6)
O2—C1—O4^i^	125.7 (4)	C40—C39—H39	119.6
O2—C1—C20	116.9 (4)	C24—C39—H39	119.6
O4^i^—C1—C20	117.3 (4)	C38—C34—C26	120.8 (6)
N1—C29—C30	122.1 (6)	C38—C34—H34	119.6
N1—C29—H29	118.9	C26—C34—H34	119.6
C30—C29—H29	118.9	C17—C44—F1	119.0 (5)
O6—O6—C3	0 (10)	C17—C44—C18	122.9 (5)
C45—C25—C24	120.7 (6)	F1—C44—C18	118.0 (6)
C45—C25—H25	119.7	F3—C41—C40	118.4 (6)
C24—C25—H25	119.7	F3—C41—C45	118.7 (6)
C33—C22—C21	120.0 (6)	C40—C41—C45	122.9 (6)
C33—C22—H22	120.0	C37—C38—C34	118.9 (5)
C21—C22—H22	120.0	C37—C38—H38	120.5
C39—C24—C25	118.8 (5)	C34—C38—H38	120.5
C39—C24—C19	120.9 (5)	C43—C42—C13	118.3 (6)
C25—C24—C19	120.2 (5)	C43—C42—H42	120.9
C20—C19—C23	119.0 (5)	C13—C42—H42	120.9
C20—C19—C24	122.6 (5)	C41—C40—C39	118.4 (6)
C23—C19—C24	118.4 (5)	C41—C40—H40	120.8
O6—C3—O6	0.0 (5)	C39—C40—H40	120.8
O6—C3—O3	125.4 (4)	C37—C36—C35	117.4 (6)
O6—C3—O3	125.4 (4)	C37—C36—H36	121.3
O6—C3—C4	118.8 (4)	C35—C36—H36	121.3
O6—C3—C4	118.8 (4)	C38—C37—F4	119.2 (5)
O3—C3—C4	115.7 (4)	C38—C37—C36	123.4 (6)
C11—C10—C13	118.2 (5)	F4—C37—C36	117.4 (6)
C11—C10—C9	120.6 (5)	C12—C43—C42	123.5 (6)
C13—C10—C9	121.0 (5)	C12—C43—F2	118.7 (6)
C35—C26—C34	117.2 (6)	C42—C43—F2	117.8 (6)
C35—C26—C21	121.9 (5)	C22—C33—C23	121.4 (6)
C34—C26—C21	120.9 (5)	C22—C33—H33	119.3
C22—C21—C20	119.5 (5)	C23—C33—H33	119.3
C22—C21—C26	118.9 (5)	C36—C35—C26	122.4 (5)
C20—C21—C26	121.6 (4)	C36—C35—H35	118.8
C9—C4—C5	119.8 (4)	C26—C35—H35	118.8
C9—C4—C3	119.1 (4)	C31—C32—C30	117.8 (6)
C5—C4—C3	121.1 (4)	C31—C32—H32	121.1
C7—C8—C9	121.1 (5)	C30—C32—H32	121.1
C7—C8—H8	119.5	C28—C31—C32	120.3 (6)
C9—C8—H8	119.5	C28—C31—H31	119.9
C42—C13—C10	120.5 (6)	C32—C31—H31	119.9
C42—C13—H13	119.8	C32—C30—C29	119.2 (6)
C10—C13—H13	119.8	C32—C30—H30	120.4
C6—C7—C8	120.5 (5)	C29—C30—H30	120.4
C6—C7—H7	119.8	C47—O7—C48	113.1 (5)
C8—C7—H7	119.8	O7—C47—C46	108.7 (5)
C16—C14—C15	118.2 (5)	O7—C47—H47A	110.0
C16—C14—C5	120.9 (5)	C46—C47—H47A	110.0
C15—C14—C5	120.7 (5)	O7—C47—H47B	110.0
C44—C18—C15	118.2 (5)	C46—C47—H47B	110.0
C44—C18—H18	120.9	H47A—C47—H47B	108.3
C15—C18—H18	120.9	C47—C46—H46A	109.5
C44—C17—C16	118.3 (6)	C47—C46—H46B	109.5
C44—C17—H17	120.8	H46A—C46—H46B	109.5
C16—C17—H17	120.8	C47—C46—H46C	109.5
C6—C5—C4	118.9 (5)	H46A—C46—H46C	109.5
C6—C5—C14	118.0 (5)	H46B—C46—H46C	109.5
C4—C5—C14	123.0 (4)	O7—C48—C49	107.3 (6)
C19—C20—C21	120.3 (5)	O7—C48—H48A	110.3
C19—C20—C1	120.3 (5)	C49—C48—H48A	110.3
C21—C20—C1	119.2 (5)	O7—C48—H48B	110.3
C8—C9—C4	118.9 (5)	C49—C48—H48B	110.3
C8—C9—C10	117.9 (5)	H48A—C48—H48B	108.5
C4—C9—C10	123.0 (4)	C48—C49—H49A	109.5
C14—C16—C17	121.7 (6)	C48—C49—H49B	109.5
C14—C16—H16	119.1	H49A—C49—H49B	109.5
C17—C16—H16	119.1	C48—C49—H49C	109.5
C18—C15—C14	120.6 (5)	H49A—C49—H49C	109.5
C18—C15—H15	119.7	H49B—C49—H49C	109.5
C14—C15—H15	119.7		

Symmetry code: (i) −*x*+1, −*y*+1, −*z*+1.

### Hydrogen-bond geometry (Å, º)

**Table:**

*D*—H···*A*	*D*—H	H···*A*	*D*···*A*	*D*—H···*A*
O5—H5···O6	0.84	1.87	2.602 (5)	145
